# Archaeal S-layer glycoproteins: post-translational modification in the face of extremes

**DOI:** 10.3389/fmicb.2014.00661

**Published:** 2014-11-26

**Authors:** Lina Kandiba, Jerry Eichler

**Affiliations:** Department of Life Sciences, Ben Gurion University of the Negev, Beersheva, Israel

**Keywords:** Archaea, lipid modification, post-translational modification, protein glycosylation, S-layer glycoprotein

## Abstract

Corresponding to the sole or basic component of the surface (S)-layer surrounding the archaeal cell in most known cases, S-layer glycoproteins are in direct contact with the harsh environments that characterize niches where Archaea can thrive. Accordingly, early work examining archaeal S-layer glycoproteins focused on identifying those properties that allow members of this group of proteins to maintain their structural integrity in the face of extremes of temperature, pH, and salinity, as well as other physical challenges. However, with expansion of the list of archaeal strains serving as model systems, as well as growth in the number of molecular tools available for the manipulation of these strains, studies on archaeal S-layer glycoproteins are currently more likely to consider the various post-translational modifications these polypeptides undergo. For instance, archaeal S-layer glycoproteins can undergo proteolytic cleavage, both *N*- and *O*-glycosylation, lipid-modification and oligomerization. In this mini-review, recent findings related to the post-translational modification of archaeal S-layer glycoproteins are considered.

Although Archaea are now recognized as denizens of an enormous range of environments, they remain best known in their capacities as extremophiles, namely organisms able to thrive in some of the most physically challenging settings on the planet. In direct contact with these often hostile surroundings, the archaeal cell surface must not only maintain its integrity but also must carry out a variety of normal physiological functions. In Bacteria, the cell boundary consists of membranes and a peptidoglycan-based cell wall together with other polysaccharide-based molecules (e.g., lipopolysaccharide, teichoic acid) and proteins (Braun, 1975; Lugtenberg and Van Alphen, 1983; Raetz et al., 2007), in many cases comprising a surface (S)-layer (Fagan and Fairweather, 2014). By contrast, the cell wall in Archaea tends to be much simpler. Apart from a number of documented examples (König, 2001), the S-layer, in many cases comprising a single protein species but not always (Peters et al., 1995; Grogan, 1996; Veith et al., 2009), corresponds to the sole cell wall structure (Eichler, 2003; Albers and Meyer, 2011). Studies from several groups studying different Archaea have shown that the S-layer glycoprotein is not just a standardized building block used to generate the two-dimensional lattice of the S-layer but rather that S-layer glycoproteins undergo a variety of post-translational modifications. In this mini-review, recent findings concerning such processing of archaeal S-layer glycoproteins are considered.

## DIFFERENCES IN THE SUGAR COATING

The S-layer glycoprotein of the haloarchaeon *Halobacterium salinarum* offered the first example of *N*-glycosylation in a domain other than the Eukarya (Mescher and Strominger, 1976a). This observation led to a flurry of biochemical activity aimed at describing the composition of *N*-linked glycans decorating the *Hbt. salinarum* S-layer glycoprotein and their biosynthesis (cf. Lechner and Wieland, 1989). However, the lack of sufficient genetic tools for manipulating this and other archaeal species shown to contain glycosylated S-layer proteins (Sumper et al., 1990; Brockl et al., 1991; Karcher et al., 1993) stood in the way of gaining detailed information into such post-translational modification of this protein. Since then, the sequencing of a growing list of archaeal genomes, the development of techniques for manipulating the genetic content of numerous strains and the analytical power of mass spectrometry have been combined to help clear obstacles encountered by earlier studies of S-layer glycoprotein *N*-glycosylation.

Genomic analyses point both to the presence of S-layer glycoproteins and *N*-glycosylation machineries in almost all sequenced Archaea (Magidovich and Eichler, 2009; Albers and Meyer, 2011; Kaminski et al., 2013a). Still, the majority of research on archaeal S-layer glycoprotein *N*-glycosylation to date has focused on *Methanococcus voltae*, *Methanococcus maripaludis*, *Sulfolobus acidocaldarius*, and *Haloferax volcanii* (for recent review, see Jarrell et al., 2014). In each of these species, genes involved in the assembly and attachment of N-linked glycans and often their protein products have been studied. Yet, apart from *S. acidocaldarius*, where *N*-glycosylation is essential for cell survival (Meyer and Albers, 2014), the elimination of such protein processing seemingly has limited impact on the organism (Abu-Qarn and Eichler, 2006; Chaban et al., 2006; VanDyke et al., 2009). As such, one can ask why Archaea devote such a significant number of genes to this post-translational modification. Recent studies on *Hfx. volcanii* S-layer glycoprotein have begun to shed light on this point.

The *Hfx. volcanii* S-layer glycoprotein contains seven putative *N*-glycosylation sites (Sumper et al., 1990). Of these, Asn-13 and Asn-83 are modified by a pentasaccharide comprising a hexose, two hexuronic acids, a methyl ester of hexuronic acid and a mannose (Abu-Qarn et al., 2007; Guan et al., 2010; Magidovich et al., 2010). However, when *Hfx. volcanii* cells are grown in medium containing 1.75 M NaCl (“low salt” conditions) rather than 3.4 M NaCl (“high salt” conditions), Asn-498 is modified by a distinct glycan comprising a sulfated hexose, two hexoses and a rhamnose (Guan et al., 2012). Indeed, the same glycan had been reported earlier as bound to dolichol phosphate in *Hfx. volcanii* grown in the presence of 1.25 M NaCl (Kuntz et al., 1997), the lipid carrier that serves as the platform for *N*-glycan assembly in this and other Archaea (Lechner et al., 1985; Guan et al., 2010; Calo et al., 2011). As such, it would appear that the *Hfx. volcanii* S-layer glycoprotein undergoes differential *N*-glycosylation as a function of environmental salinity. While it remains to be defined how such differential S-layer glycoprotein *N*-glycosylation translates into an appropriate response to changes in surrounding salt levels, the path involved in the biogenesis of the so-called “low-salt” tetrasaccharide has been revealed (Kaminski et al., 2013b). Unexpectedly, the cluster of genes involved does not include an obvious oligosaccharyltransferase, namely that enzyme responsible for transferring a glycan from its lipid carrier to select Asn residues of target proteins (Mohorko et al., 2011). The observation that AglB, the only known archaeal oligosaccharyltransferase (Abu-Qarn and Eichler, 2006; Chaban et al., 2006), is not involved in “low-salt” tetrasaccharide attachment implies the existence of a novel yet undefined enzyme as serving this role (Kaminski et al., 2013b).

It is possible that *N*-glycosylation of the *Hfx. volcanii* S-layer glycoprotein is even more complicated still. It was recently reported that the Asn-732 position is modified by a sulfoquinovose-hexose-based glycan, *N*-linked via a chitobiose core (Parente et al., 2014). Moreover, the composition of this glycan was modified in response to the absence or presence of a membrane-localized rhomboid protease. The presence of such a glycan in *Hfx. volcanii* is surprising, given this organism does not contain a homolog of *S. acidocaldarius* Agl3 (Meyer et al., 2011), a UDP-sulfoquinovose synthase responsible for converting UDP-glucose and sodium sulfite into UDP-sulfoquinovose, the activated form of this sugar that is presumably used in *S. acidocaldarius* and presumably *Hfx. volcanii N*-glycosylation. It should also be noted that Asn-732 is found in the same C-terminal region as a cluster of *O*-glycosylated threonine residues (Sumper et al., 1990) and a lipid anchor (see below). This suggests that post-translational modification of the *Hfx. volcanii* S-layer glycoprotein C-terminal region is a complex event that requires the orchestrated involvement of numerous protein processing pathways.

Unlike *Hfx. volcanii*, which must cope with an environment characterized by molar concentrations of salt, *S. acidocaldarius* is a thermophile that grows optimally at 75–80^°^C and pH 2–3 (Brock et al., 1972). Possibly due to the challenges presented by its surroundings, not only is *N*-glycosylation essential in *S. acidocaldarius* (Meyer and Albers, 2014) but at least one of the glycoproteins comprising the S-layer in this species (SlaA) presents an extremely high number of *N*-glycosylation sites. The 1,395 amino acid-long protein contains 31 potential sites for *N*-glycosylation scattered throughout the polypeptide, translating to an *N*-glycosylation site every 45 residues on average, with the highest densely of such sites being seen in the C-terminal quarter of the protein (Peyfoon et al., 2010). In the region spanning Lys-1004 to Gln-1395, nine of the 11 potential *N*-glycosylation sites were experimentally verified as being charged with a tri-branched hexasaccharide comprising a glucose, a mannose, two *N*-acetylglucosamines and a sulfoquinovose, an unusual sugar routinely found in chloroplasts and photosynthetic bacteria (Zahringer et al., 2000; Peyfoon et al., 2010). Indeed, a tallying of the number of putative *N*-glycosylation sites in 20 different archaeal S-layer glycoproteins reveals that the S-layer glycoproteins of thermo(acido)philes can contain up to 20-fold more such sites than do S-layer glycoproteins in species isolated from other growth conditions (Jarrell et al., 2014). Based on this comparison, it was proposed that such high densities of *N*-glycosylation sites reflect the need for a rigid and stable cell wall to cope with the challenges of elevated temperatures and acidity encountered by thermo(acido)philic Archaea.

The importance of S-layer glycoprotein glycosylation was also demonstrated in recent work linking the activity of a transcription factor controlling the expression of genes involved in sugar metabolism with S-layer glycoprotein glycosylation and hence, with the maintenance of cell shape in *Hbt. salinarum* (Todor et al., 2014). TrmB binds to the promoters of over 110 genes encoding products involved in various metabolic processes in response to glucose concentrations. Yet, *Hbt. salinarum* does not catabolize glucose, cannot use glucose as the sole carbon or energy source and does not actively transport glucose from the media (Gochnauer and Kushner, 1969; Severina et al., 1990). As such, it was proposed that TrmB activity ensures that sufficient amounts of glucose and other monosaccharides are available for S-layer glycoprotein glycosylation. S-layer glycoprotein glycosylation is directly related to *Hbt. salinarum* maintaining its rod-like shape, with a loss of *N*-glycosylation leading to the appearance of round cells (Mescher and Strominger, 1976b). Hence, TrmB activity is linked to *Hbt. salinarum* shape and by extension to cell growth, since this process requires the presence of sufficient fully processed S-layer glycoprotein.

Finally, *O*-glycosylation, where the glycan is linked to the hydroxyl group of Ser or Thr residues, has been reported for both the *Hbt. salinarum* and the *Hfx. volcanii* S-layer glycoproteins (Mescher and Strominger, 1976a; Sumper et al., 1990). In both proteins, Thr-rich regions adjacent to the predicted membrane-spanning domain of the protein are modified with galactose–glucose disaccharides. Still today, nothing is known of the pathways responsible for *O*-glycosylation in Archaea.

## HANGING ON BY A LIPID

Just as S-layer glycoproteins have served as tractable reporters of archaeal protein glycosylation, they have also been central to our understanding of lipid modification in Archaea, namely the covalent linkage of lipid-based groups to a polypeptide chain. Relying on various biochemical approaches, it was shown that the S-layer glycoproteins of *Hbt. salinarum* and *Hfx. volcanii* undergo lipid modification (Kikuchi et al., 1999; Konrad and Eichler, 2002). However, it is only of late that insight into the process of such lipid modification has been provided.

Analysis of the deduced amino acid sequence of the *Hfx. volcanii* S-layer glycoprotein (Sumper et al., 1990) predicts the existence of a 20-residue-long C-terminal membrane-spanning domain, thought to anchor the protein within the membrane. At the same time, it was shown that EDTA treatment leads to the release of the S-layer glycoprotein into the surrounding growth medium (Cline et al., 1989). Solving the paradox of how an apparently integral membrane protein could be solubilized by divalent cation chelation began with studies showing incorporation of radiolabeled polyprenol precursors into the *Hfx. volcanii* S-layer glycoprotein. This observation led to the conclusion that the protein is subjected to magnesium-dependent processing associated with lipid modification (Eichler, 2001; Konrad and Eichler, 2002). A decade later, a combination of sequential solubilization steps, native gel electrophoresis and mass spectrometry pointed to the existence of two distinct sub-populations of the S-layer glycoprotein, the first corresponding to an EDTA-solubilized pool anchored to the membrane via a covalently linked archaetidic acid lipid anchor and the second representing detergent-solubilized pool anchored to the membrane likely via the C-terminal membrane-spanning domain (Kandiba et al., 2013). Both S-layer glycoproteins were shown to be *N*-glycosylated.

In the same period, it was proposed that the Pro-Gly-Phe motif found just upstream of the presumed C-terminal membrane-spanning domain of the *Hfx. volcanii* and *Hbt. salinarum* S-layer glycoproteins is processed similarly as a comparable motif found in certain membrane-linked Gram-positive bacterial proteins (Haft et al., 2012). In Bacteria, this motif is cleaved by a transpeptidase called an exosortase and the released protein is linked to the cell wall via a waiting lipid anchor. Accordingly, genome sequence analysis predicted the existence of an archaeal version of exosortase, termed archaeosortase A (ArtA). Subsequent genetic and biochemical work confirmed not only the existence of ArtA but also its ability to cleave the *Hfx. volcanii* S-layer glycoprotein at the C-terminal Pro-Gly-Phe motif described above (Abdul Halim et al., 2013).

**FIGURE 1:**
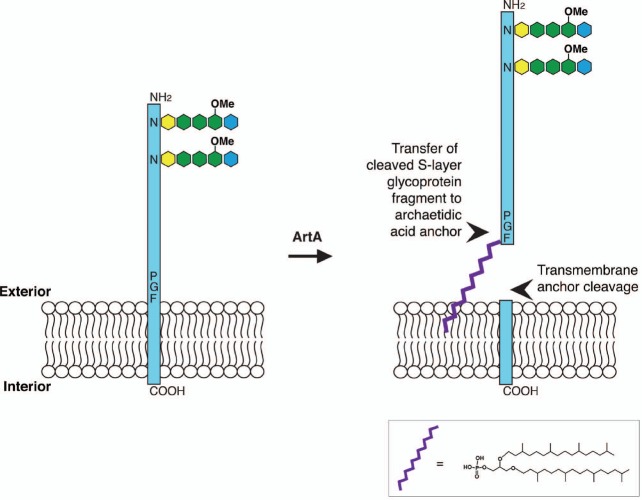
**Schematic depiction of the proposed *Hfx. volcanii* S-layer glycoprotein lipid modification process.** In *Hfx. volcanii*, the S-layer glycoprotein is synthesized with a C-terminal membrane-spanning domain. ArtA cleaves the protein at a PGF motif immediately upstream of the transmembrane domain. The cleaved S-layer glycoprotein fragment is transferred to a waiting archaetidic acid anchor, schematically depicted in purple (detailed structure provided). Alternatively, attachment of the lipid anchor could proceed protein cleavage. In either case, two S-layer glycoprotein populations appear. Such lipid modification transpires following N-glycosylation of the protein. Of the seven putative N-glycosylation sites, Asn-13 and Asn-83 are modified by a pentasaccharide comprising a hexose (yellow), three hexuronic acids (green; the last a methyl ester of hexuronic acid) and a mannose (blue). The temporal relation between lipid modification and O-glycosylation of a cluster of Thr residues found above the cleavage site (not shown) remains to be determined.

Together, the results of these recent studies argue that in *Hfx. volcanii* (and likely in *Hbt. salinarum* as well), the S-layer glycoprotein is initially synthesized with a C-terminal membrane-spanning domain. This precursor is cleaved by ArtA and the processed S-layer glycoprotein is transferred to a waiting archaetidic acid anchor lipid anchor in a magnesium-dependent manner. Still, as only selected aspects of this hypothesized pathway (Figure 1) have been demonstrated, further experiments await.

## CONCLUSION

Corresponding to the building block of the S-layer, the outermost limit of the archaeal cell surface, S-layer glycoproteins are not only in direct contact with the harsh environments Archaea can inhabit but are also amongst the first archaeal proteins to encounter any changes in those environments. Post-translational modification of S-layer glycoproteins offer a rapid and reversible response to such changes. Soon, ongoing efforts in laboratories around the world will not only provide further insight into the pathways recruited for these protein processing events but will also hopefully reveal how such modifications affect S-layer structure and stability. Indeed, with the availability of high resolution structures of archaeal *S*-glycoproteins (Arbing et al., 2012), it will be possible to obtain detailed understanding of the contributions of post-translational modification to S-layer architecture not only as a function of environment but also of growth stage and other physiological conditions.

## AUTHOR CONTRIBUTIONS

All authors made substantial contributions to the acquisition, analysis, and interpretation of data described in this report. All authors critically reviewed the report and approved the final version. All authors agree to be accountable for all aspects of the work in ensuring that questions related to the accuracy or integrity of any part of the work are appropriately investigated and resolved.

### Conflict of Interest Statement

The authors declare that the research was conducted in the absence of any commercial or financial relationships that could be construed as a potential conflict of interest.
